# The relationship between sarcopenia and breast cancer: mechanistic pathways and clinical relevance

**DOI:** 10.3389/fcell.2026.1731822

**Published:** 2026-07-09

**Authors:** Yuheng Wu, Hongliang Cao, Mengxin Li, Jinghui Hong, Hongxu Zhou, Xiaochuan Gao, Dong Song

**Affiliations:** 1 Department of Breast Surgery, General Surgery Center, The First Hospital of Jilin University, Changchun, China; 2 Department of Urology II, The First Hospital of Jilin University, Changchun, China

**Keywords:** body composition, breast cancer, myokine, sarcopenia, survival prognosis, systemic inflammation, therapeutic toxicity

## Abstract

Breast cancer is the most frequently diagnosed malignancy among women worldwide and one of the leading causes of cancer-related mortality. Despite advances in screening and therapeutic strategies, its prevention and treatment continue to face considerable challenges due to pronounced tumor heterogeneity, therapeutic resistance, and treatment-related toxicities. Sarcopenia, a syndrome characterized by progressive loss of skeletal muscle mass (SMM) and function, is increasingly prevalent in patients with breast cancer and has been consistently associated with adverse clinical outcomes. In this review, we systematically summarize epidemiological evidence linking sarcopenia to breast cancer and elucidate its association with increased postoperative complications, heightened toxicity from systemic therapies, and inferior survival outcomes. We further explore potential pathophysiological mechanisms underlying this association, including altered pharmacokinetics that may contribute to exacerbated drug toxicity, dysregulated myokine signaling, and chronic inflammation that is linked to tumor progression, insulin resistance, and detrimental changes in physical function and health behaviors. In addition, current evidence regarding strategies for the identification and management of sarcopenia—encompassing diagnostic assessments, pharmacological interventions, nutritional support, and exercise training—is critically evaluated. By integrating current findings, this review highlights the pivotal role of sarcopenia in the development and progression of breast cancer and provides insight into multimodal intervention strategies that may improve clinical outcomes in this population.

## 1 Introduction

Breast cancer is a common malignant tumor, with invasive ductal cancer and lobular carcinoma being prevalent histological subtypes(Board, 2002). Breast cancer remains the most frequently diagnosed cancer and the leading cause of cancer-related mortality among females, and continues to represent a major contributor to the overall cancer burden in the general population, including both sexes (Hong et al., 2023; Kim J. et al., 2025). Across most countries, the incidence of breast cancer is on the rise; this increase is especially marked in countries with a lower Human Development Index, which also faced disproportionately greater mortality (Kim J. et al., 2025). With the implementation of screening programs and the advancement of multidisciplinary treatment modalities—including surgery, radiotherapy, chemotherapy, endocrine therapy, targeted therapy, and immunotherapy—the prognosis for many patients has improved significantly (Xiong et al., 2025). However, clinical management of breast cancer still faces numerous challenges, such as tumor heterogeneity, drug resistance, adverse treatment effects, and variable treatment responses (Ji et al., 2019; Kudelova et al., 2022; Donahue et al., 2023). These issues underscore the urgent need for further research to identify new biomarkers, clarify disease mechanisms, and optimize personalized therapeutic strategies, ultimately to enhance the diagnosis and management of breast cancer.

Sarcopenia is defined as a progressive and generalized disorder of skeletal muscle, marked by an accelerated decline in muscle mass and function (Cruz-Jentoft and Sayer, 2019). The reported prevalence of sarcopenia varies considerably across studies, mainly due to differences in the diagnostic criteria applied (Sayer et al., 2024). While sarcopenia commonly occurs among older adults, it has been found to be closely associated with a range of systemic diseases, including diabetes, cirrhosis, chronic kidney disease, and various malignancies (Shachar et al., 2016; Mintziras et al., 2018; Su et al., 2019; Yang et al., 2019; Chen et al., 2022), such as breast cancer (Zhang et al., 2020). Individuals with sarcopenia exhibit higher rates of both short- and long-term mortality as well as reduced OS and progression-free survival (PFS) (Yuan and Larsson, 2023). Recent studies have demonstrated a strong association between sarcopenia and multiple clinical aspects of breast cancer. Individuals with sarcopenia are at a markedly increased risk of developing severe treatment-related toxicities during breast cancer therapy, such as myelosuppression, gastrointestinal disturbances, and peripheral neuropathy, which can significantly compromise treatment adherence, often resulting in chemotherapy dose reductions or discontinuation and ultimately diminishing therapeutic efficacy (Shachar et al., 2017b; Jang et al., 2024; Roberto et al., 2024). Furthermore, breast cancer patients with sarcopenia exhibit poorer survival outcomes, including significantly elevated risks of all-cause mortality and disease progression or recurrence (Zhang et al., 2020; Roberto et al., 2024). Early screening and identification of sarcopenia during cancer treatment, along with interventions such as exercise to prevent or reverse muscle loss, may contribute to improved overall management and prognosis in patients with breast cancer (Roberto et al., 2024; Tan et al., 2024). Regarding other body composition abnormalities, sarcopenic obesity is also significantly associated with increased all-cause mortality and higher rates of complications from both systemic therapy and surgery in patients with various solid tumors across different treatment settings (Baracos and Arribas, 2018). Similarly, obesity in cancer patients is associated with higher incidences of surgical complications and treatment-related toxicities, as well as increased risks of tumor recurrence and cancer-specific mortality (Lysaght and Conroy, 2024).

In this review, we summarize the current epidemiological and experimental evidence supporting a robust relationship between sarcopenia and breast cancer. We further explore the underlying mechanisms that may mediate this association and highlight potential strategies for targeting sarcopenia to improve the clinical management and prognosis of breast cancer. We aim to provide new insights into the complex interplay between sarcopenia and breast cancer, and to inspire novel approaches for optimizing breast cancer care. Unlike previous narrative reviews, the present review critically appraises study design biases, focuses on breast cancer-specific mechanistic pathways, and highlights evidence gaps across molecular subtypes and treatment regimens.

### 1.1 Search strategy and study selection

To comprehensively identify literature on the relationship between sarcopenia and breast cancer, we systematically searched PubMed, Web of Science, and the Cochrane Library databases from inception to June 2025. The search strategy employed Boolean operators (AND, OR) combining terms related to sarcopenia (e.g., “sarcopenia”, “skeletal muscle mass”, “muscle wasting”, “low muscle mass”, “myopenia”, “skeletal muscle density”, “skeletal muscle index”) and breast cancer (e.g., “breast cancer”, “breast carcinoma”, “breast neoplasm”). In addition, we manually screened the reference lists of the included articles to identify further relevant studies.

Original research articles and high-quality narrative reviews were included. Case reports, conference abstracts, non-English articles, and studies that did not provide sufficient data to evaluate the association between sarcopenia and breast cancer outcomes were excluded. Two authors independently screened titles, abstracts, and full texts. Disagreements were resolved through discussion or consultation with other authors. Data extraction encompassed study design, sample size, diagnostic criteria for sarcopenia, outcome measures, and main findings. Given the considerable heterogeneity in the definitions, measurement methods, and outcome reporting of sarcopenia, a formal meta-analysis was not performed. Instead, we adopted a narrative synthesis approach while critically appraising methodological limitations.

## 2 A growing body of observational findings underline the close link between sarcopenia and breast cancer

### 2.1 The prevalence of sarcopenia is significantly elevated in breast cancer patients

Multiple studies have revealed a significantly higher prevalence of sarcopenia in breast cancer patients (Table 1). The prevalence of sarcopenia varies among different cancer populations. A multicenter cross-sectional study involving 766 cancer patients found that the overall prevalence of low muscle mass (LMM) was 69.1% (Raynard et al., 2022). In contrast, breast cancer patients exhibited a comparatively lower prevalence than other cancer types; it remained notably high at 54.3% (Raynard et al., 2022). Another meta-analysis encompassing 280 studies indicated that the prevalence of sarcopenia across different tumor types ranged from 27.2% to 61.0%, reaching 32% in breast cancer patients (Surov and Wienke, 2022). A further meta-analysis reported the prevalence of low skeletal muscle index (SMI) and low skeletal muscle density (SMD) in common solid tumors, with breast cancer patients showing a low SMI prevalence of 34% and a low SMD prevalence of 55.3% (McGovern et al., 2021). The prevalence of sarcopenia in breast cancer is significantly higher than in healthy populations. A cross-sectional study including 122 breast cancer patients and 80 healthy women found that approximately 13.9% of the breast cancer patients were diagnosed with sarcopenia, whereas no cases were identified in the control group (Morlino et al., 2022). The prevalence is markedly increased in metastatic breast cancer compared to stages I-III. One meta-analysis of 12 studies reported a sarcopenia prevalence of 32.5% in stage I-III breast cancer patients (Jang et al., 2024), and another meta-analysis of 14 studies on metastatic breast cancer found an overall prevalence of 41.6% (Jang et al., 2025). Cancer treatment may also influence the prevalence. A retrospective study of 111 patients with metastatic breast cancer demonstrated a significant increase in sarcopenia prevalence from the time of metastatic diagnosis to the initiation of treatment (including chemotherapy, endocrine therapy, and targeted therapy); however, no significant change was observed between the start of treatment and the 3-month follow-up (Camilleri et al., 2024). These findings collectively indicate that sarcopenia is highly prevalent in breast cancer patients and is associated with disease progression and therapeutic interventions.

**TABLE 1:** Prevalence of sarcopenia in breast cancer.

Country	First author	Year	Sample size	Diagnostic criteria	Conclusion	References
France	Bruno Raynard	2022	766	Male SMI < 55 cm^2^/m^2^; Female SMI < 39 cm^2^/m^2^	The prevalence of low muscle mass was 69.1% in cancer patients and 54.3% in breast cancer patients.	Raynard et al. (2022)
Multiple countries	Alexey Surov	2022	280 studies	Varying across studies: Male SMI < 47–63.94 cm^2^/m^2^; Female SMI < 23–29.1 cm^2^/m^2^	The prevalence of sarcopenia varied widely across cancer types (27.2%–61.0%), with a prevalence of 32% in breast cancer.	Surov and Wienke (2022)
Multiple countries	Josh McGovern	2021	160 studies	Varying across studies: Low SMI – Male SMI ≤ 25.66–55.4 cm^2^/m^2^, Female SMI ≤ 21.73–46.4 cm^2^/m^2^; Low SMD – Male SMD < 22–44.4 HU, Female SMD < 23.5–41 HU	The prevalence of low SMI was 34% and low SMD was 55.3% in breast cancer.	McGovern et al. (2021)
Italy	Delia Morlino	2022	122	Based on EWGSOP2 criteria	The prevalence of sarcopenia was 13.9% in breast cancer patients, with no cases in the control group.	Morlino et al. (2022)
Multiple countries	Min Kyeong Jang	2024	17 studies	Varying across studies: SMI < 38.5–41 cm^2^/m^2^	The prevalence of sarcopenia was 32.5% in patients with Stage I–III breast cancer.	Jang et al. (2024)
France	Géraldine M. Camilleri	2024	111	SMI < 39 cm^2^/m^2^	The prevalence of sarcopenia increased significantly from diagnosis to the start of treatment in metastatic breast cancer.	Camilleri et al. (2024)
Multiple countries	Min Kyeong Jang	2025	14 studies	Varying across studies: SMI < 38–41 cm^2^/m^2^	The overall prevalence of sarcopenia was 41.6% in metastatic breast cancer.	Jang et al. (2025)

Abbreviation: EWGSOP, European Working Group on Sarcopenia in Older People; HU, Hounsfield Unit; SMD, Skeletal Muscle Density; SMI, Skeletal Muscle Index

In summary, sarcopenia is consistently more prevalent in breast cancer patients than in healthy controls, especially in metastatic disease, yet reported estimates vary widely (14%–55%) owing to heterogeneous diagnostic criteria. Currently, no subtype-specific prevalence data exist for different molecular subtypes of breast cancer.

### 2.2 Sarcopenia is associated with increased surgical complication rates in breast cancer

Multiple studies have indicated a correlation between sarcopenia and an increased incidence of surgical complications (Table 2). A retrospective study of 157 patients found that in those undergoing tissue expander placement following mastectomy, the ratio of low skeletal muscle mass (SMM) to total adipose tissue index (TATI) was an independent risk factor for delayed wound healing (Nakamura et al., 2020). Research on another surgical procedure indicated that in patients undergoing autologous breast reconstruction, those with sarcopenia were more prone to delayed flap healing, accompanied by an increased risk of reoperation and prolonged hospital stay (Pittelkow et al., 2020). Another retrospective analysis of 88 patients who underwent total mastectomy showed a significantly higher incidence of flap necrosis in sarcopenic patients (Yabe et al., 2021). However, a study involving 208 patients receiving abdominally based microvascular breast reconstruction found that pre-existing sarcopenia did not significantly increase the risk of surgical complications (Broyles et al., 2020). Another study analyzing 682 breast cancer patients who underwent various surgical procedures also did not observe an association between sarcopenia and surgical complications (Aleixo et al., 2023b).

**TABLE 2:** Association between sarcopenia and postoperative complications in breast cancer.

Country	First author	Year	Sample size	Diagnostic criteria	Conclusion	References
Japan	Hideharu Nakamura	2020	157	No study-specific low SMI cut-off value was defined; SMI was analyzed as a continuous variable.	A lower ratio of SMI to TATI was an independent risk factor for complications and delayed wound healing following expander-based reconstruction.	Nakamura et al. (2020)
United States	Justin M Broyles	2020	208	SMI < 38.5 cm^2^/m^2^	Sarcopenia showed no significant association with minor or major surgical complications.	Broyles et al. (2020)
United States	Eric M Pittelkow	2020	103	SMI ≤ 38.5 cm^2^/m^2^	Patients with sarcopenia demonstrated significantly increased rates of delayed flap healing, return to the operating room, and prolonged hospital stay.	Pittelkow et al. (2020)
Japan	Sakiko Yabe	2021	88	Psoas Muscle Index (PMI, cm^2^/m^2^) was used, with cut-off values of PMI < 6.36 cm^2^/m^2^ for males and PMI < 3.92 cm^2^/m^2^ for females.	Sarcopenia was associated with a significantly higher incidence of flap necrosis after total mastectomy.	Yabe et al. (2021)
United States	Gabriel Francisco Pereira Aleixo	2023	682	SMM was measured using BIS. SMI = SMM/ height^2^ (kg/m^2^). Sarcopenia was diagnosed as SMI ≤ 6.75 kg/m^2^.	No association was found between sarcopenia and the incidence of adverse surgical outcomes across any procedure type.	Aleixo et al. (2023b)

Abbreviation: BIS, Bioelectrical Impedance Spectrometry; PMI, Psoas Muscle Index; SMI, Skeletal Muscle Index; SMM, Skeletal Muscle Mass; TATI, total adipose tissue index

Overall, evidence is inconsistent, some studies show increased complications with sarcopenia while others do not, likely due to heterogeneity in definitions and procedures. No molecular subtype-specific data are available, as none of the studies stratified outcomes by HR/HER2 status.

### 2.3 Sarcopenia is associated with increased incidence of adverse reactions to systemic therapy in breast cancer

Several recent studies have focused on the association between sarcopenia and adverse reactions to systemic therapy in breast cancer (Supplementary Table S1). A retrospective study of 151 patients with early-stage breast cancer demonstrated that, among patients treated with anthracycline and cyclophosphamide followed by taxane-based chemotherapy, reduced baseline SMI and SMD were significantly associated with grade 3–4 chemotherapy toxicities (including hematological and gastrointestinal toxicities) and hospitalization (Shachar et al., 2017b). Furthermore, the composite index skeletal muscle gauge (SMG; SMG = SMI × SMD) was more effective than individual indices in identifying high-risk patients (Shachar et al., 2017b). Another larger study (n = 338) found that low SMD was independently associated with adverse events, including chemotherapy dose reduction, early treatment discontinuation, and hospitalization, in a multivariate model (Aleixo et al., 2020). Studies in Asian populations have yielded similar findings; for instance, during epirubicin plus cyclophosphamide chemotherapy, patients with sarcopenia had a significantly higher incidence of grade ≥3 laboratory adverse events (LAEs) (Ueno et al., 2020). A Korean study found that sarcopenia was associated with an increased risk of anemia induced by neoadjuvant chemotherapy (Jang et al., 2023). Four systematic reviews and meta-analyses have consolidated evidence showing that baseline sarcopenia significantly increases the risk of grade ≥3 chemotherapy toxicities (Aleixo et al., 2019; Jang et al., 2024; Roberto et al., 2024; Lewis et al., 2025). However, heterogeneity in SMI/SMD thresholds and measurement methods across studies underscores the need for further standardization in both clinical research and practice. Beyond SMI and SMD, body composition changes assessed by other methods have also been linked to chemotherapy-related adverse reactions. For example, lower skeletal muscle area (SMA) at the T11 vertebral level was associated with increased systemic paclitaxel concentration, while prolonged infusion time may reduce supra-therapeutic exposure and peripheral neuropathy (Hertz et al., 2022). Another study used CT to quantify the pectoralis muscle index (PMI) and investigate its correlation with anthracycline-related cardiotoxicity. The results indicated that higher PMI was associated with a lower risk of major adverse cardiac events (Toama et al., 2022). Additionally, sarcopenia diagnosed via bioelectrical impedance spectroscopy (BIS) was also significantly associated with higher chemotherapy toxicity, dose reductions or delays, and hospitalization (Aleixo et al., 2023a).

In metastatic breast cancer, most studies similarly reveal a correlation between sarcopenia and adverse reactions to chemotherapy. In a cohort receiving first-line taxane therapy, patients with sarcopenia had significantly higher rates of grade 3–4 toxicities, hospitalization, and dose reduction or treatment delays compared to those without sarcopenia (Shachar et al., 2017a). Similar outcomes were observed in a metastatic cohort treated with capecitabine (Prado et al., 2009). A prospective study in metastatic breast cancer suggested that the presence of sarcopenia at any point during a 6-month study period was associated with an increased risk of grade ≥2 treatment-related toxicities (Delrieu et al., 2021). However, some studies have reported conflicting results. For instance, a French multicenter study (the SCAN study) found no association between sarcopenia and adverse events in its metastatic breast cancer subgroup, possibly because not all enrolled patients received chemotherapy or because the chemotherapeutic agents used had lower toxicity profiles (Deluche et al., 2022). Another study focusing on older adults with advanced cancers showed no significant association between sarcopenia and treatment toxicity, potentially because toxicity in elderly patients is more strongly influenced by organ aging than by sarcopenia (Versteeg et al., 2018).

In the context of endocrine therapy, multiple studies also indicate a link between sarcopenia and treatment toxicity. A multicenter retrospective study of 305 patients with early-stage HR + breast cancer showed that sarcopenia was significantly associated with early discontinuation or switching of endocrine therapy due to toxicity (Saraf et al., 2024). Another study using BIS for sarcopenia assessment yielded similar results, indicating that sarcopenia was correlated with increased endocrine-related side effects and therapy discontinuation due to adverse events (Aleixo et al., 2022). However, two studies involving metastatic HR + breast cancer patients treated with endocrine therapy combined with CDK4/6 inhibitors did not observe any association between sarcopenia and treatment-related toxicity (Yücel et al., 2024; Cavdar et al., 2025).

The relationship between sarcopenia and adverse reactions has also been explored in breast cancer targeted therapy. Among HR + metastatic breast cancer patients with PIK3CA mutations, those with lower SMG had a higher risk of grade ≥2 toxicities. Lower SMD and lower visceral adipose tissue (VAT) were associated with alpelisib-related hyperglycemia, rash, and hospitalization. These findings suggest that sarcopenia may limit the safety and effective delivery of PI3K inhibitor therapy (Shachar et al., 2024).

Several study cohorts have included patients receiving various systemic therapies, which may better reflect real-world clinical scenarios, as breast cancer patients often receive multiple systemic treatments concurrently or sequentially. One study of 85 early-stage breast cancer patients receiving standard interventions—including neoadjuvant or adjuvant chemotherapy, hormone therapy, and targeted therapy—found that a lower SMI at diagnosis was significantly associated with a higher incidence of grade 4 neutropenia (Kang et al., 2024). In contrast, no association between treatment toxicity and body composition was observed in an HR- metastatic breast cancer cohort undergoing multiple systemic therapies (Sheean et al., 2021).

Consistent meta-analytic evidence confirms that baseline sarcopenia is a robust predictor of grade ≥3 chemotherapy toxicity, dose reductions, hospitalization, and treatment discontinuation in breast cancer, across both early and metastatic stages. However, while some CDK4/6 inhibitor and endocrine therapy studies have been restricted to specific molecular subtypes (Sheean et al., 2021; Saraf et al., 2024; Yücel et al., 2024; Cavdar et al., 2025), these are inclusion criteria rather than comparative analyses across subtypes. Prospective data stratified by disease stage, molecular subtype, and treatment regimen remain scarce, and standardized diagnostic criteria for sarcopenia are urgently needed.

### 2.4 Impact of sarcopenia on breast cancer prognosis

The overall survival (OS) rate of breast cancer has progressively improved with advances in screening and precision treatment. Sarcopenia, a syndrome reflecting the body’s overall nutritional and metabolic status, exerts an independent and profound impact on breast cancer prognosis, a conclusion supported by multiple studies (Supplementary Table S2).

Numerous systematic reviews and meta-analyses consistently indicate that sarcopenia or low SMM/SMD in breast cancer patients is significantly associated with a higher risk of mortality, establishing robust overall evidence for its role as an independent adverse prognostic factor (Zhang et al., 2020; Dai et al., 2024; Jang et al., 2024). However, one study yielded divergent results; while simultaneously assessing SMM and SMD, it found that sarcopenia did not significantly affect survival or recurrence outcomes in breast cancer patients. Potential reasons for this discrepancy include the lack of standardized SMI thresholds and variations in breast cancer treatment regimens, which also influence survival (Deng et al., 2024). Similarly, a multicenter study initially found no association between LMM and prognosis (Ballinger et al., 2023). Still, after adjusting the SMI threshold to 36 cm^2^/m^2^, LMM was correlated with poorer OS and PFS (Ballinger et al., 2023), suggesting that SMI thresholds require further determination through prospective studies.

Beyond the most commonly used SMI threshold, sarcopenia defined by other body composition measurements also predicts breast cancer prognosis. Two large-scale studies indicated that the composite phenotype of “LMM combined with high adiposity” carries the highest risk. Patients with high total adipose tissue (TAT) and those with sarcopenia both exhibited higher overall mortality, body mass index (BMI) alone failed to predict mortality, underscoring the importance of combined assessment of muscle and fat (Caan et al., 2018; Song et al., 2018) Several other metrics assessed via BIA or CT have shown significant associations with OS, distant metastasis-free survival (DMFS), or PFS, including the appendicular skeletal muscle mass index (ASMI) (Liu et al., 2022), total abdominal muscle area (TAMA) (Huh et al., 2020), PMI (Huang et al., 2022), muscle fat infiltration or intermuscular adipose tissue (IMAT) area index (Deluche et al., 2018), intramuscular adipose tissue content corrected (IMAT-C) (Aktas et al., 2025), and SMI at the T12 level (Gu et al., 2025). Furthermore, combining body composition measurements with various inflammatory indices plays a crucial role in predicting survival for breast cancer patients. One study demonstrated that patients with both sarcopenia and a high systemic immune-inflammation index (SII) (Tang et al., 2022) had significantly worse OS compared to those with sarcopenia alone. The combined predictive value of SMI with the neutrophil-to-lymphocyte ratio (NLR) (Hua et al., 2020) and pectoralis muscle area (PMA) with the lymphocyte-to-monocyte ratio (LMR) (Song et al., 2022) has also been reported.

In metastatic breast cancer, the scenario differs. Low SMD appears to be a stronger indicator of survival risk than SMA. SMG, which integrates SMI and SMD, is a marginally significant predictor of adverse events and OS (Rier et al., 2017; Shachar et al., 2017a). Myosteatosis, diagnosed based on SMD and BMI, showed a significant adverse association with 2-year survival (Sheean et al., 2021). Two studies that did not incorporate SMD assessment, relying solely on SMI or appendicular lean mass (ALM) to diagnose sarcopenia, failed to find an association between sarcopenia and mortality risk in metastatic breast cancer (Zhang et al., 2020; Camilleri et al., 2024), further supporting the potential importance of SMD. Sarcopenia, defined by the EWGSOP2 criteria, independently predicted short-term overall mortality at 6 months (Martinez-Tapia et al., 2023). A systematic review offered several explanations for the phenomenon where SMI fails to predict outcomes in metastatic breast cancer, including high heterogeneity across studies, varying SMI thresholds, the inherent high heterogeneity of metastatic breast cancer itself, and its treatments. Additionally, the role of sarcopenia in metastatic disease might differ from other cancers or be masked by stronger prognostic factors such as tumor burden, inflammatory status, or malnutrition (Jang et al., 2025).

Studies focusing on specific metastatic sites also suggest a link between sarcopenia and prognosis. One study analyzing patients with breast cancer brain metastases treated with gamma knife radiosurgery found that sarcopenia was associated with increased mortality at multiple follow-up time points (Sim et al., 2024). Another cohort with brain metastases identified temporal muscle thickness as an independent predictor of survival (Furtner et al., 2017). In a cohort with breast cancer bone metastases, low psoas muscle density, but not psoas muscle area, was associated with lower median OS (Yao et al., 2022). Research on a mixed tumor cohort (including breast cancer) with spinal metastases showed that both psoas muscle area and the psoas muscle/vertebral body ratio significantly predicted OS, independent of tumor histology and gender (Zakaria et al., 2020).

Similar findings have been observed in metastatic breast cancer cohorts undergoing different systemic therapies. In HER2-positive metastatic breast cancer patients receiving first-line pertuzumab/trastuzumab, higher baseline abdominal subcutaneous fat index and total abdominal fat index were associated with shorter PFS (Palleschi et al., 2022). Interestingly, the impact of sarcopenia on PFS was not significant, possibly due to the milder adverse effects of targeted therapy compared to chemotherapy (Palleschi et al., 2022). In an HR+/HER2− metastatic breast cancer cohort treated with CDK4/6 inhibitors, patients with sarcopenia had reduced OS and PFS (Cavdar et al., 2025), and SMD was a better predictor of treatment response and PFS/OS than SMI (Kim H. et al., 2025). VAT and BMI also predicted treatment response (Kripa et al., 2022; Yücel et al., 2024), with higher visceral fat index and visceral fat density associated with better PFS (Franzoi et al., 2020), highlighting the superiority of a multi-faceted body composition assessment.

The Miller–Payne system is a standard method for evaluating the response to neoadjuvant therapy in breast cancer. However, it relies on post-operative pathological assessment, making it challenging to adjust treatment plans preoperatively for patients with a poor response. Multiple studies suggest that sarcopenia can predict treatment responsiveness, potentially aiding in the pre-therapeutic prediction of neoadjuvant therapy efficacy. In a cohort of triple-negative breast cancer patients receiving neoadjuvant chemotherapy, pre-treatment sarcopenia was associated with a significantly reduced pathological complete response (pCR) rate, and combining this with MRI radiomics further enhanced predictive capability (Guo et al., 2024). Another study in locally advanced breast cancer showed an increased prevalence of sarcopenia after neoadjuvant chemotherapy, and patients without sarcopenia were more likely to achieve pCR (Karaca et al., 2024). BMI is also related to efficacy; high BMI combined with sarcopenia was associated with reduced pCR rates and poorer follow-up outcomes (Del Fabbro et al., 2012). However, this study also found that among patients with normal BMI, those with sarcopenia had a better prognosis than those without, possibly due to receiving relatively higher chemotherapy doses and exhibiting better tolerance (Del Fabbro et al., 2012). This indicates that sarcopenia assessment should be combined with BMI for improved accuracy.

Multiple studies demonstrate that the negative impact of sarcopenia on breast cancer patients is long-term, cumulative, and severely affects quality of life and long-term prognosis. Two extensive studies showed that patients with pre-diagnosis sarcopenia had significantly decreased OS during long-term follow-up (Villaseñor et al., 2012; Su et al., 2023). A systematic review indicated that sarcopenia adversely affects both short-term (<5 years) and long-term (≥5 years) mortality, with a more pronounced effect in long-term follow-up, suggesting a cumulative negative impact (Dai et al., 2024). One study conducted body composition assessments at diagnosis and at three- and 5-year post-diagnosis and found a continuous decline in muscle-related indicators, such as SMA and SMI (Kang et al., 2024). A similar trend was reported in metastatic breast cancer, where the prevalence of sarcopenia increased significantly from the time of diagnosis to the initiation of treatment (Camilleri et al., 2024), further supporting the notion of an ongoing adverse effect.

Sarcopenia, characterized by low muscle strength and LMM, severely impacts quality of life (Xia et al., 2020). For breast cancer patients with sarcopenia, the situation is more severe. One study showed that sarcopenic phenotypes (e.g., decreased muscle strength) in women with breast cancer were strongly associated with a high risk of fractures (de Almeida Marques Bernabé et al., 2022). Reduced PMI in older breast cancer patients is highly correlated with frailty, further exacerbating the decline in quality of life and increasing the care burden (Kızılarslanoğlu et al., 2023). A study assessing the association between symptoms like dyspnea, pain, cough, and fatigue and sarcopenia in patients with malignant pleural effusions (a group including breast cancer patients) found that sarcopenic patients had worse health-related quality of life and functional status, indicating that sarcopenia exacerbates cancer-related symptoms and functional decline (Rodríguez-Torres et al., 2019). Furthermore, an umbrella review revealed that sarcopenia significantly increases the risk of dysphagia, cognitive impairment, fractures, falls, hospitalization, and all-cause mortality in older cancer patients, and is also associated with higher levels of proteinuria, depression, and various metabolic diseases (Xia et al., 2020).

### 2.5 Diagnostic heterogeneity limits comparability across studies

In existing studies, the definitions and cut-off values used to diagnose sarcopenia vary considerably, which is an important factor limiting comparability across studies. To systematically address this issue, we summarized the major diagnostic criteria, measurement methods, sources of cut-off values, and prospective validation status used in breast cancer-related studies (Supplementary Table S3).

EWGSOP2 identifies low muscle strength as the key feature of sarcopenia and confirms the diagnosis by combining it with low muscle quantity or quality. This framework is more suitable for geriatric, nutritional, and rehabilitation assessment. In contrast, breast cancer studies more commonly use CT-based metrics, particularly L3-SMI and SMD. These imaging-based measures can be conveniently derived from staging or follow-up scans, but they usually do not capture functional parameters such as handgrip strength or gait speed. Even when CT-SMI is used, the applied thresholds are not consistent across studies. In Western cohorts, commonly used female L3-SMI cut-offs vary, and some studies further apply BMI-stratified thresholds. Similar heterogeneity is also present in Asian cohorts, where some studies adopted previously proposed cut-offs, whereas others derived study-specific thresholds using ROC curves, AUC, median values, or other cohort-based methods. Although such cohort-derived thresholds may help explore prognostic relevance in specific populations, they should not be directly generalized as universal diagnostic standards. In addition, the anatomical level and muscle group selected for measurement may also affect the results. Total skeletal muscle area at the L3 level is generally considered to provide a reasonable estimate of whole-body muscle mass. However, because abdominal CT is not always available in patients with breast cancer, alternative measures have also been used, including SMI at other anatomical levels, pectoralis muscle area or index, psoas muscle area or index, and psoas muscle density. These regional muscle measurements are not directly interchangeable with L3-SMI. Similarly, SMD, LMA, IMAT, and IMAT-C mainly reflect muscle quality or fatty infiltration rather than muscle quantity alone.

Such diagnostic heterogeneity may partly explain the inconsistent findings reported across studies. Future studies should clearly report the anatomical level, muscle group, source of cut-off values, and whether sex-, BMI-, or ethnicity-stratified thresholds were applied. The predictive performance of different criteria should also be compared in prospective breast cancer cohorts.

Multiple systematic reviews and meta-analyses consistently demonstrate that sarcopenia is significantly associated with reduced OS and increased mortality risk in breast cancer patients, with a cumulative negative effect over the long term. However, the diagnostic criteria for sarcopenia remain highly heterogeneous, leading to considerable variability in effect sizes across studies. The available evidence is derived predominantly from retrospective or cross-sectional studies, whereas prospective cohorts and interventional trials are extremely scarce. Notably, TNBC and HER2+ subtypes appear overrepresented in existing meta-analyses, whereas the HR+/HER2– subtype, which accounts for the majority of breast cancer cases, is underrepresented. This imbalance may bias the pooled effect estimates and limit the generalizability of prognostic conclusions. A few studies have restricted enrollment to specific molecular subtypes (Sheean et al., 2021; Palleschi et al., 2022; Guo et al., 2024; Cavdar et al., 2025), but such restrictions serve as inclusion criteria rather than direct comparative analyses between different subtypes. One exploratory study (Rier et al., 2017) suggested that sarcopenia might have stronger prognostic significance in HER2-positive and TNBC, however, this was a *post hoc* analysis with a limited sample size, and its findings should be considered hypothesis-generating only. Therefore, there is an urgent need to establish standardized diagnostic and assessment criteria for sarcopenia and to conduct high-quality prospective studies with prospective stratification by molecular subtype, disease stage, and treatment regimen, in order to clarify the independent prognostic value of sarcopenia and to provide a robust basis for precision risk management in breast cancer.

## 3 Several possible pathways mediate the association between sarcopenia and breast cancer

The observational findings summarized above consistently link sarcopenia to adverse breast cancer outcomes. However, these associations do not imply causality, as reverse causation and residual confounding cannot be excluded. In this section, we discuss potential biological mechanisms that may explain the observed relationships, including pharmacokinetic alterations, myokine dysregulation and chronic inflammation, insulin resistance, and impaired physical function.

### 3.1 Sarcopenia influences pharmacokinetics and increases the risk of drug-related adverse reactions

Chemotherapy is one of the most critical systemic treatments for breast cancer. Adjuvant chemotherapy helps eliminate micrometastatic disease, reduces the risk of recurrence and metastasis, and significantly improves DFS and OS (Anampa et al., 2015). Neoadjuvant chemotherapy increases the rate of breast-conserving surgery and is as effective as adjuvant chemotherapy in terms of long-term survival and recurrence-free survival (Early Breast Cancer Trialists’ Collaborative Group, 2018). In metastatic breast cancer, chemotherapy can significantly alleviate symptoms, reduce tumor-related discomfort, and improve quality of life (Johnston, 2011). However, chemotherapeutic agents are associated with numerous adverse events that severely impact treatment adherence (Al-Mahayri et al., 2020). Several studies have indicated that lower drug clearance and higher drug concentrations are linked to an increased incidence of adverse reactions to epirubicin and paclitaxel (Sandström et al., 2005; Aldaz et al., 2023; Tang et al., 2025), suggesting that pharmacokinetic parameters are predictors of adverse effects.

**FIGURE 1:**
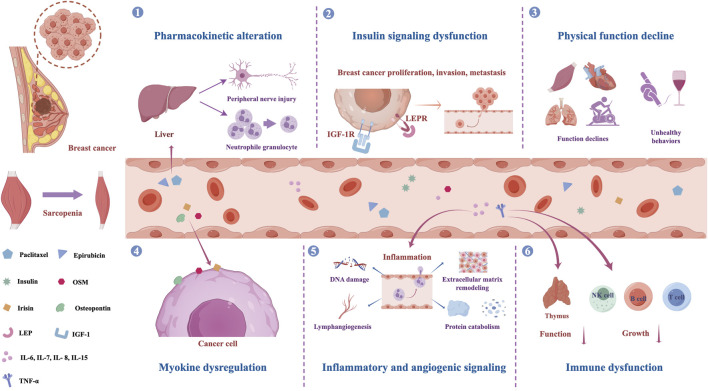
Potential mechanisms by which sarcopenia influences breast cancer prognosis. Sarcopenia may affect breast cancer outcomes through six interrelated mechanisms: (1) Impairs the metabolism of paclitaxel and epirubicin, increasing drug toxicity. This has been directly demonstrated in breast cancer studies. (2) Induces insulin resistance and activates the PI3K/AKT/mTOR pro-cancer pathway. This mechanism is largely extrapolated from preclinical models and awaits validation. (3) Reduces muscle strength, exercise capacity, and cardiorespiratory function while promoting unhealthy behaviors, leading to poor postoperative outcomes. Observational studies support this association, but causality remains inferred. (4) Causes dysregulation of oncostatin M, irisin, and osteopontin, which may facilitate tumor progression. Direct evidence exists in breast cancer research. (5) Disrupts IL-6/IL-8/IL-15 signaling, promoting inflammation and tumor development; chronic inflammation may further aggravate muscle loss, forming a vicious cycle. This mechanism is partially extrapolated. (6) Impairs NK cell, lymphocyte, and thymic function via IL-6, IL-7, IL-15, and TNF-α, delaying wound healing. The IL-6-mediated NK cell impairment is supported by animal and human studies (Pedersen et al., 2016), but has not been directly validated in sarcopenic breast cancer patients. This mechanism is considered hypothetical.

Direct evidence from breast cancer patients indicates that sarcopenia influences pharmacokinetics and increases the risk of drug-related toxicity (Figure 1). One observational study in breast cancer patients found that the paclitaxel volume of distribution was significantly correlated with SMA at the thoracic level (Hertz et al., 2022). LMM was associated with a reduced volume of distribution, resulting in elevated C_max_ levels and an increased risk of peripheral neuropathy; prolonging the infusion time helped mitigate this adverse effect (Hertz et al., 2022). Another prospective study showing that each 1 kg increase in lean body mass (LBM) was associated with approximately a 19% increase in epirubicin clearance, suggesting that LBM is a predictor of drug exposure and toxicity (Prado et al., 2011). Furthermore, an observational study in Asian breast cancer patients found that abdominal fat volume was significantly positively correlated with the area under the concentration-time curve (AUC) of doxorubicin, whereas muscle volume showed no significant association, suggesting that the pharmacokinetic profiles of different drugs may depend on the distribution of body components (Wong et al., 2014).

Inferences from general sarcopenia research suggest that patients with sarcopenia often have elevated levels of pro-inflammatory cytokines, such as TNF-α and C-reactive protein (CRP). These inflammatory mediators may further affect pharmacokinetics and increase the risk of adverse reactions by modulating the activity of drug-metabolizing enzymes (Aleixo et al., 2022). It is noteworthy that current clinical chemotherapy dosing remains primarily based on body surface area (BSA). This method overlooks significant interpatient variations in body composition, particularly in patients with sarcopenia, potentially leading to dose overestimation, which may explain their higher incidence of toxicity and risk of treatment failure. Some researchers have proposed that LBM may more accurately predict drug dosage than BSA or total body weight, highlighting the need for future dose individualization (Morgan and Bray, 1994). In summary, sarcopenia affects drug metabolism processes and increases the incidence of drug-related adverse reactions.

### 3.2 Sarcopenia-associated myokine dysregulation and immune dysfunction are linked to poorer breast cancer prognosis and may play a role in delayed postoperative wound healing

Skeletal muscle is not only an organ of locomotion but also an endocrine organ. Pedersen et al. proposed that cytokines and other peptides produced, expressed, and released by muscle fibers, which exert autocrine, paracrine, or endocrine effects, should be classified as myokines (Pedersen and Febbraio, 2012). More than 650 myokines have been identified, such as IL-6, IL-7, IL-15, Oncostatin M (OSM), and Irisin. These myokines are involved not only in regulating muscle-specific processes such as myogenesis, energy metabolism, and inflammatory responses but also in facilitating functional crosstalk between skeletal muscle and other organs—including the liver, adipose tissue, bone, brain, and immune system—via the muscle–organ axis (Severinsen and Pedersen, 2020). In cancer, myokines mediate their effects on disease outcomes through multiple pathways, including direct tumor interactions, amelioration of chronic inflammation, and immunomodulatory functions (Figure 1) (Severinsen and Pedersen, 2020).

It has been demonstrated that multiple myokines can directly interact with breast cancer cells or correlate with tumor progression. OSM suppresses breast cancer cell proliferation and thereby promotes apoptosis (Hojman et al., 2011). Further observational studies in breast cancer patients have shown that Irisin levels are negatively correlated with breast cancer risk, yet are higher in stage III patients compared to those in earlier stages, suggesting its role may shift with disease progression (Provatopoulou et al., 2015). Osteopontin levels increase over the course of breast cancer and, in patients with metastatic disease, are significantly associated with decreased survival (Bramwell et al., 2006). The loss of muscle mass is associated with myokine dysregulation, which may promote tumor progression.

Clinical evidence directly indicates that both sarcopenic patients and breast cancer patients exhibit elevated markers of inflammatory response. A meta-analysis including 3,072 patients with sarcopenia and 8,177 controls showed that sarcopenia was significantly associated with elevated serum CRP levels, though not significantly with IL-6 or TNF-α (Bano et al., 2017). Studies focusing on breast cancer populations have found increased NLR in patients with low SMD (Kim H. et al., 2025). Chronic inflammation promotes tumor progression by stimulating angiogenesis, causing DNA damage, and remodeling the extracellular matrix; it also induces protein catabolism, further exacerbating muscle loss and forming a vicious cycle (Coussens and Werb, 2002; Bartsch and Nair, 2006; Costamagna et al., 2015; Mazzuca et al., 2018), which may lead to poor prognosis and increased postoperative complications in breast cancer patients (Nakamura et al., 2020; Yabe et al., 2021). Indirect evidence from non-breast-cancer populations further supports the association between sarcopenia and inflammation. Exercise-induced IL-6 activates anti-inflammatory pathways by promoting the secretion of IL-1 receptor antagonist and IL-10, while suppressing IL-1β signaling and TNF-α synthesis (Severinsen and Pedersen, 2020). However, physical inactivity, common among sarcopenic individuals, dysregulates the secretion of IL-6, IL-8, and IL-15, thereby enhancing systemic inflammation (Pratesi et al., 2013).

Regarding immune dysregulation, there is limited but existing direct evidence in breast cancer. IL-15, produced by skeletal muscle, is essential for maintaining the number and function of NK cells and CD8^+^ T cells (Fehniger and Caligiuri, 2001). Dysregulated IL-15 signaling and activation have been observed in the muscle of breast cancer patients (Bohlen et al., 2018), potentially leading to immune dysfunction and impaired prognosis. This mechanism may also reduce resistance to postoperative wound infection, delay healing (Nakamura et al., 2020), and increase the incidence of skin flap necrosis (Yabe et al., 2021). Currently, most mechanistic insights are still derived from animal models or non-breast-cancer population. Research in an animal model demonstrated that exercise-induced IL-6 of muscular origin inhibits tumor growth by enhancing NK cell tumor-killing capacity (Pedersen et al., 2016). This protective process is likely impaired in sarcopenic patients, contributing to poorer outcomes. Similarly, IL-7, also secreted by skeletal muscle, supports lymphocyte development and thymic function, playing a vital role in maintaining T-cell and B-cell homeostasis (Nelke et al., 2019). Exercise-induced increases in IL-7 enhance its thymoprotective effects, improving immune and muscle function (Duggal et al., 2018). Elevated NLR, observed in sarcopenia (Kim H. et al., 2025), may promote tumor growth via neutrophil-mediated suppression of lymphocyte activity and T-cell responses (De Larco et al., 2004). Furthermore, TNF-α levels are elevated in sarcopenia (Bian et al., 2017) and are known to promote migration, invasion, and poor prognosis in breast cancer (Liu et al., 2016). In summary, sarcopenia may lead to dysregulation of myokines such as IL-6, IL-15, IL-7, and TNF-α, elevate NLR, and cause immune dysfunction; however, direct evidence in sarcopenic breast cancer patients remains limited.

### 3.3 Sarcopenia leads to abnormal insulin signaling, affects breast cancer prognosis, and mediates drug resistance

Numerous studies have shown that sarcopenia is correlated with insulin resistance and abnormalities in insulin signaling pathways, which may in turn influence the prognosis of breast cancer patients (Figure 1). Sarcopenia is often accompanied by low SMD and increased skeletal muscle adipose tissue, both of which are independently associated with insulin resistance (Miljkovic et al., 2013). Insulin resistance refers to a reduced response to insulin in peripheral tissues (muscle, adipose tissue, liver), leading to increased insulin secretion to maintain glucose homeostasis, thereby resulting in hyperinsulinemia. If left uncorrected, long-term insulin resistance can ultimately progress to type 2 diabetes (Yee et al., 2020). Multiple cohort studies and meta-analyses have shown that type 2 diabetes significantly increases the risk of mortality in breast cancer patients (Barone et al., 2008), and elevated fasting insulin levels are also associated with an increased risk of all-cause mortality in cancer patients (Dankner et al., 2012). A cross-sectional study indicated that sarcopenia is associated with dysglycemia in individuals with insulin resistance and obesity (Srikanthan et al., 2010).

A large number of *in vitro* cellular and animal model studies have directly revealed the correlation between insulin signaling pathways and breast cancer. Insulin and insulin-like growth factor-1 (IGF-1) share receptor pathways, activating the PI3K/AKT/mTOR pathway, which enhances breast cancer cell proliferation, anti-apoptotic capacity, glycolytic metabolic reprogramming, and invasive and metastatic potential (Xue and Hemmings, 2013; Yee et al., 2020). Furthermore, insulin activates PI3K/AKT signaling and downstream NFκB, leading to the production of inflammatory cytokines and an increased M2/M1 macrophage ratio (Liu et al., 2017), thereby promoting tumor immune escape and enhancing breast cancer aggressiveness (Mosser and Edwards, 2008; Dietze et al., 2018). Leptin and IGF-1 signaling trans-activate the epidermal growth factor receptor (EGFR), significantly enhancing the invasive and migratory capabilities of breast cancer cells (Saxena et al., 2008). In ER-positive cells, leptin can also attenuate the anti-proliferative effects of tamoxifen and activate key signaling pathways (ERK1/2, STAT3) involved in endocrine resistance (Yom et al., 2013; Sánchez-Jiménez et al., 2019). Additionally, a study on Ewing sarcoma revealed that IGF-1R activation is one of the pathways contributing to resistance to CDK4/6 inhibitors (Guenther et al., 2019).

In summary, existing studies have separately established associations between sarcopenia and insulin resistance/signaling abnormalities, as well as between insulin signaling pathway abnormalities and aggressive breast cancer phenotypes. However, direct evidence linking all three components in a causal chain remains lacking. Future studies should aim to validate this pathway using mediation analysis or prospective interventional trials in cohorts of breast cancer patients with sarcopenia.

### 3.4 Sarcopenia impairs physiological function and promotes unhealthy lifestyles, contributing to poor postoperative outcomes and prognosis in breast cancer

Multiple studies indicate that body composition and quality of life are closely associated with breast cancer (Figure 1). Impairment of muscular functions leads to lifestyle changes, including reduced physical activity, inadequate dietary intake, and the emergence of unhealthy habits such as smoking and alcohol consumption (Villaseñor et al., 2012). These factors are all associated with poor breast cancer prognosis (Rock and Demark-Wahnefried, 2002; Irwin et al., 2005). Evidence from an umbrella review shows that obesity, alcohol consumption, and smoking increase the risk of developing breast cancer, while physical activity reduces this risk (Hoxha et al., 2024). For breast cancer survivors, factors such as obesity, physical inactivity, and alcohol consumption are associated with a lower health-related quality of life and contribute to an increased risk of type 2 diabetes, cardiovascular disease, second primary cancers, as well as breast cancer recurrence and mortality (Hoedjes et al., 2025). However, no study has directly quantified the independent impact of sarcopenia-induced behavioral changes on prognosis in a cohort of breast cancer patients with sarcopenia.

In sarcopenic patients, the reduction in both the number and quality of muscle fibers may lead to decreased muscle strength, diminished exercise endurance, and impaired balance function, thereby triggering adverse clinical events such as frailty, prolonged hospitalization, and falls. Retrospective studies have directly shown that poor nutrition, low body weight, and frailty in sarcopenic patients may affect postoperative wound healing (Nakamura et al., 2020) and increase the incidence of skin flap necrosis (Yabe et al., 2021), contributing to a higher rate of surgical complications. Skeletal muscle also plays a vital role in respiration and in maintaining cardiac output (Cederholm et al., 2011). When muscle mass declines, these functions are simultaneously compromised, further exacerbating the loss of physiological capacity.

Collectively, sarcopenia contributes to poor outcomes in breast cancer patients through multiple pathways: by impairing drug metabolism, promoting inflammation and immune dysregulation, disrupting insulin signaling, and influencing lifestyle behaviors. However, current evidence is derived mainly from cross-sectional/retrospective and small-sample studies, with varying thresholds and measurement protocols for SMI and SMD. Future research should further explore body composition-based precision dosing, the crosstalk and dynamic changes in myokine-inflammatory markers and insulin pathways, and the conduct of prospective experimental studies.

## 4 Clinical outcomes associated with sarcopenia-directed interventions for breast cancer

### 4.1 Identification and assessment of sarcopenia

**FIGURE 2:**
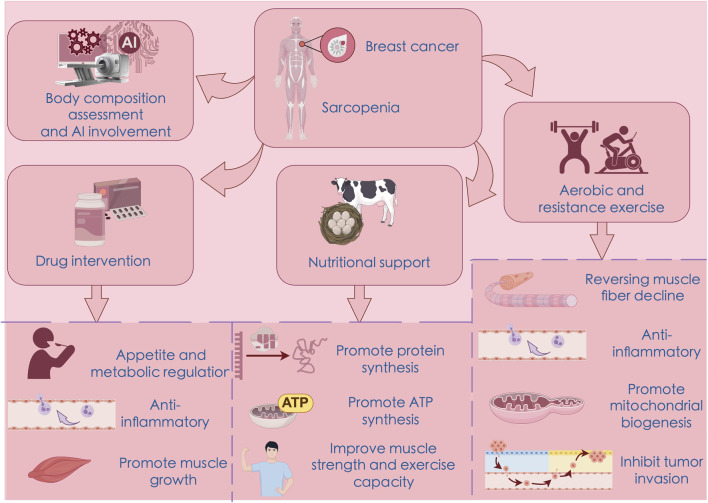
Multidimensional strategies for intervention of breast cancer-related sarcopenia and their association with clinical outcomes. Body composition assessment, facilitated by artificial intelligence (AI), enables early identification and risk stratification of sarcopenia, thereby promoting timely intervention. Pharmacological interventions primarily focus on improving appetite and metabolism, promoting muscle anabolism, inhibiting catabolism, and exerting anti-inflammatory effects. Nutritional support interventions—such as optimizing protein intake and supplementing with ω-3 fatty acids, HMB, and creatine—help promote protein and ATP synthesis and enhance muscle strength and physical performance. Aerobic and resistance training serve as core interventions, not only improving body composition and muscle strength but also conferring benefits such as reduced inflammation, metabolic improvements, and potential adjunct antitumor effects. Collectively, multidisciplinary and integrated interventions hold significant clinical value for the prevention and management of sarcopenia in breast cancer patients.

The efficient identification and assessment of malnutrition and sarcopenia are prerequisites for early intervention (Figure 2). The protocol for nutritional risk in oncology, developed by an international panel of experts in cancer-related nutrition and oncology, provides a standardized tool for the early identification, assessment, and intervention of cancer-associated malnutrition/sarcopenia across various tumor types by comprehensively evaluating body weight, dietary intake, and strength/activity levels (Muscaritoli et al., 2023).

In breast cancer, various assessment protocols for sarcopenia have been reported. Given the limited accessibility of body composition assessment techniques such as CT/DXA in clinical practice, SARC-CalF, as a sarcopenia risk screening tool, has demonstrated high specificity but low sensitivity in women with breast cancer (de Souza Mamede et al., 2025), allowing it to function as an initial screening tool. Regarding the interpretation of imaging results, the automatic body composition analyzer using computed tomography image segmentation (ABACS) software can segment muscle and adipose tissue at the L3 level with accuracy comparable to manual analysis among nonmetastatic breast cancer patients (Cheng et al., 2024), demonstrating promise for reducing analysis costs and improving efficiency. For risk stratification, the B-Score integrates measurements of low SMI, high subcutaneous adipose tissue index (SATI), and high subcutaneous adipose tissue radiodensity into a composite score, demonstrating superior performance compared with stratification based on single indicators (Cheng et al., 2024). Beyond imaging assessment, artificial intelligence (AI) has been applied to identify predictors of cancer-associated sarcopenia. Using an XGBoost model to analyze joint angle data from a Kinect-based mixed-reality system, the right knee flexion angle was identified as the most influential predictor, with squat duration and shoulder range of motion also significant (Lim and Song, 2025).

### 4.2 Pharmacological interventions for sarcopenia

Drug discovery efforts targeting cancer-associated sarcopenia primarily focus on three pathways: anabolic promotion of muscle growth, appetite/metabolic regulation, and anti-inflammation (Figure 2). AMG 745/Mu-S increased LBM and reduced fat mass in a prostate cancer population with good tolerability, suggesting potential dual “muscle-building and fat-reducing” effects (Padhi et al., 2014). The selective androgen receptor modulator enobosarm exerts tissue-selective anabolic effects on muscle and bone, improving LBM, physical performance, and insulin resistance among healthy elderly men and postmenopausal women (Dalton et al., 2011). Metformin, an effective agent for reducing insulin resistance, has been shown to decrease apelin and IL-15 levels in patients with stage I–III breast or colorectal cancer (Brown et al., 2024), and its combination with galantamine improved physical function, muscle mass, and strength in an animal model (Tezze et al., 2023). The selective melanocortin-4 receptor (MC-4R) antagonist BL-6020/979 simultaneously increased appetite, reduced energy expenditure, and improved body composition in a mouse model (Dallmann et al., 2011). Activation of nuclear factor-kappa B (NF-κB) in immune cells upregulates cytokine expression. The anti-inflammatory drugs indomethacin (IND) (Zhou et al., 2003) and pyrrolidine dithiocarbamate (PDTC) (Nai et al., 2007) have been shown to reduce NF-κB activation and TNF-α and IL-6 serum levels, thereby attenuating weight loss and muscle atrophy in animal models. Although existing studies demonstrate therapeutic effects through various mechanisms, much of the evidence remains confined to animal models or non-breast-cancer populations, and sufficient robust clinical evidence from breast cancer-specific randomized controlled trials is lacking.

### 4.3 Nutritional support interventions for sarcopenia

Multiple studies indicate that optimizing protein intake combined with resistance training is an effective strategy for managing sarcopenia (Figure 2). Compared to routine intake, increased protein consumption significantly improves SMI in elderly women with sarcopenia (Muscariello et al., 2016). A systematic review demonstrated that protein supplementation combined with muscle-strengthening exercises simultaneously enhances LBM, lower limb strength, and walking capacity in elderly patients with a high risk of sarcopenia or frailty (Liao et al., 2019). Furthermore, supplementation with ω-3 fatty acids enhances muscle protein synthesis and SMM, thereby providing additional benefit for sarcopenia (Di Girolamo et al., 2014). However, a systematic review (Ries et al., 2012), noted that ω-3 fatty acids did not demonstrate significant benefits in patients with various advanced cancers, including breast cancer, possibly due to substantial clinical heterogeneity among the participants and the advanced stage of disease at the time of enrollment. Additionally, β-hydroxy β-methylbutyrate (HMB) not only increases muscle protein synthesis but also reduces muscle protein breakdown; systematic reviews suggest its benefits for improving muscle mass and function in patients with various types of cancers (Prado et al., 2022). Combined supplementation with HMB, arginine, and lysine for 12 weeks increased fat-free mass and muscle strength while boosting protein synthesis in healthy elderly women (Flakoll et al., 2004). Creatine promotes ATP production in muscles, and existing research has proven that short-term supplementation significantly increases muscle mass and strength (Rawson and Venezia, 2011). However, a study involving breast cancer patients showed no significant improvement in muscle strength after just 7 days of creatine supplementation (Parsowith et al., 2024), suggesting that the efficacy of nutritional supplementation might be lower in breast cancer patients compared to healthy individuals, potentially necessitating combined intervention strategies for enhanced effectiveness. Another study appears to support this inference: dietary counseling alone was insufficient to alleviate sarcopenia in breast cancer patients undergoing endocrine therapy, and needed to be combined with exercise interventions like resistance training to achieve substantial improvements in body composition and function (Pedersini et al., 2024). However, it should be noted that most of the aforementioned nutritional strategies (e.g., ω-3 fatty acids, HMB, creatine) lack sufficient robust clinical evidence from breast cancer-specific randomized controlled trials and cannot yet be routinely recommended for breast cancer patients.

### 4.4 Exercise interventions for sarcopenia

A growing body of evidence highlights the critical role of exercise interventions in preventing and treating sarcopenia in cancer patients (Figure 2). Regarding evidence from systematic reviews, a meta-analysis of 7 studies found that exercise not only promotes muscle synthesis but also improves cancer patient prognosis by mitigating sarcopenia (Cao et al., 2022). Another meta-analysis encompassing 11 studies further confirmed that resistance training is a recommended intervention for reducing body fat, increasing muscle mass and strength, and enhancing physical function (Tan et al., 2024).

Numerous clinical studies indicate that various types of cancer populations benefit from exercise. A prospective cohort study of patients with metastatic breast cancer found that 6 months of aerobic exercise did not significantly increase muscle cross-sectional area or density, but it did slow muscle loss (Delrieu et al., 2021). This holds positive clinical significance, particularly in the context of a disease and its treatment that leads to substantial muscle wasting (Delrieu et al., 2021). In patients with early-stage breast cancer, a multicenter randomized controlled trial demonstrated that resistance exercise training (RET) effectively reversed sarcopenia and improved quality of life and fatigue (Adams et al., 2016). In contrast, aerobic exercise training (AET) was more advantageous in reducing body fat (Adams et al., 2016). Furthermore, another clinical trial showed that individualized, telephone-guided strength and aerobic training significantly slowed the decline in physical function, as quantified by the short physical performance battery score, in older cancer patients during follow-up (Arrieta et al., 2019). A non-randomized prospective study also suggested that implementing aerobic and resistance training during endocrine therapy for breast cancer helps improve bone-related metrics and body composition (Hojan et al., 2013). The benefits of exercise for cancer patients extend beyond improvements in body composition. A systematic review found that interventions combining resistance and endurance exercise can lead to sustained improvements in fatigue, depression, cardiorespiratory fitness, muscle strength, and quality of life in breast cancer patients (Mok et al., 2022). A randomized controlled trial in breast cancer patients indicated that the benefits of exercise vary among individuals: among those undergoing chemotherapy, patients with normal body weight, who were premenopausal and younger, were more likely to derive greater benefit from higher doses of exercise, underscoring the importance of personalized exercise regimens (Courneya et al., 2014).

The benefits of exercise are also validated at the tissue and molecular levels. With aging, the number of skeletal muscle type II fibers and their associated satellite cells both decrease significantly; RET can reverse this process, thereby improving muscle regeneration potential (Verdijk et al., 2014). Combined aerobic and resistance exercise interventions not only improve metabolic syndrome and sarcopenic obesity but also significantly reduce biomarkers of metabolism and inflammation, such as insulin, IGF-1, leptin, and adiponectin (Dieli-Conwright et al., 2018a). Both AET and RET can also suppress systemic inflammation by reducing the M1/M2 macrophage ratio and decreasing IL-6 and TNF-α levels (Dieli-Conwright et al., 2018b). Animal studies suggest that exercise promotes mitochondrial biogenesis, enhances antioxidant gene expression, reduces intramuscular stressor levels, and induces tumor cells to differentiate towards a less invasive phenotype, indicating that exercise may possess both anti-sarcopenic and potential adjunct anti-tumor effects (Mader et al., 2022).

Based on the evidence presented above and recommendations from international guidelines (Ligibel et al., 2022; Weimann et al., 2025), the clinical management of sarcopenia in breast cancer can be approached at two levels: currently implementable recommendations and research priorities. For current clinical practice, early screening using tools such as SARC-CalF and evidence-based protocol is recommended, along with CT-based body composition assessment (L3 level) for surgical candidates, routine prescription of aerobic and resistance exercise during chemotherapy and rehabilitation, and optimization of protein intake with nutritional supplementation. Regarding research priorities, efforts should focus on standardizing sarcopenia diagnostic criteria and validating ethnic-specific cut-off values, clinically validating body composition-based precision chemotherapy dosing models, conducting randomized controlled trials of nutritional supplements and pharmacological agents in breast cancer-specific populations. Notably, the ESPEN 2025 guideline (Weimann et al., 2025) on clinical nutrition in surgery has incorporated sarcopenia diagnosis and prehabilitation into its recommendations and endorses CT as the gold standard for body composition assessment. The ASCO 2022 guideline (Ligibel et al., 2022), while affirming the value of exercise for improving functional outcomes, has not made a definitive recommendation for improving cancer control outcomes.

## 5 Conclusions and future directions

Although current research has confirmed a significant association between sarcopenia and adverse clinical outcomes in breast cancer, the causal relationship and underlying mechanisms remain to be fully elucidated. It is important to note that most of the evidence cited in this review derives from cross-sectional or retrospective studies. Distinct from prior overviews, the present work critically examines methodological biases that limit causal inference and identifies actionable research priorities. Because breast cancer progression and its associated antitumor treatments may themselves induce skeletal muscle loss, these study designs inherently struggle to rule out reverse causality, potentially creating a spurious association between sarcopenia and poor prognosis. Furthermore, the diagnostic criteria for sarcopenia vary considerably across studies, highlighting an urgent need for standardization and harmonization.

Beyond these general limitations, other biases warrant explicit discussion. Selection bias is likely present: most studies collected CT imaging data, a design that may overlook asymptomatic or mildly symptomatic patients who lack clinical indications for CT scans. Residual confounding is pervasive, as few studies adequately adjusted for tumor stage, treatment regimen, systemic inflammation, nutritional status, or physical activity level—factors that may be independently associated with both muscle loss and cancer outcomes. Therefore, the observed associations should be interpreted with caution. Ongoing clinical trials, such as the study evaluating resistance exercise intervention for sarcopenia in breast cancer patients undergoing neoadjuvant chemotherapy (KCT0008961) and the trial investigating sarcopenia as a predictor of treatment-related toxicity (NCT06274268), may provide higher-level evidence in the future.

The potential mechanisms involve the interplay of multiple pathways, including disturbances in drug metabolism, systemic inflammation, immune dysregulation, and insulin resistance. Future research should prioritize large-scale prospective studies and randomized controlled trials to establish causality, while also delving deeper into the molecular mechanisms linking these two conditions, with a particular focus on the role of myokines and the interactions among various inflammatory mediators. Efforts are also needed to refine sarcopenia assessment methods, implement individualized precision dosing based on body composition, and develop multimodal intervention strategies that integrate exercise training, nutritional support, and pharmacological approaches. The ultimate goal is to incorporate comprehensive sarcopenia management into the holistic treatment framework for breast cancer, which may help improve long-term patient outcomes.

## Supplementary material

SUPPLEMENTARY TABLE S1 Association between sarcopenia and systemic treatment-related toxicities in breast cancer.

SUPPLEMENTARY TABLE S2 Association between sarcopenia and prognosis in breast cancer.

SUPPLEMENTARY TABLE S3 Comparison of sarcopenia diagnostic criteria and body composition metrics used in breast cancer-related studies.
